# A New Synthetic Route to Original Sulfonamide Derivatives in 2-Trichloromethylquinazoline Series: A Structure-Activity Relationship Study of Antiplasmodial Activity

**DOI:** 10.3390/molecules17078105

**Published:** 2012-07-05

**Authors:** Nicolas Primas, Pierre Verhaeghe, Anita Cohen, Charline Kieffer, Aurélien Dumètre, Sébastien Hutter, Sylvain Rault, Pascal Rathelot, Nadine Azas, Patrice Vanelle

**Affiliations:** 1 Laboratoire de Pharmacochimie Radicalaire, Faculté de Pharmacie, Institut de Chimie Radicalaire UMR CNRS 7273, Aix-Marseille Univ, 27 Boulevard Jean Moulin, 13385 Marseille cedex 05, France; Email: nicolas.primas@univ-amu.fr (N.P.); pierre.verhaeghe@univ-amu.fr (P.V.); anita.cohen@univ-amu.fr (A.C.); charline.kieffer@ap-hm.fr (C.K.); pascal.rathelot@univ-amu.fr (P.R.); 2 Infections Parasitaires, Transmission, Physiopathologie et Thérapeutique, UMR MD3, Faculté de Pharmacie, Aix-Marseille Univ, 27 Boulevard Jean Moulin, 13385 Marseille Cedex 05, France; Email: aurelien.dumetre@univ-amu.fr (A.D.); sebastien.hutter@univ-amu.fr (S.H.); nadine.azas@univ-amu.fr (N.A.); 3 Centre d’Etudes et de Recherche sur le Médicament de Normandie, UPRES EA 4258, FR CNRS 3038 INC3M, UFR des Sciences Pharmaceutiques, Université de Caen Basse-Normandie, Boulevard Becquerel, 14032 Caen Cedex, France; Email: sylvain.rault@unicaen.fr

**Keywords:** quinazoline, trichloromethyl group, sulfonamide, microwaves, antiplasmodial activity

## Abstract

We report herein a simple and efficient two-step synthetic approach to new 2-trichloromethylquinazolines possessing a variously substituted sulfonamide group at position 4 used to prepare new quinazolines with antiparasitic properties. Thus, an original series of 20 derivatives was synthesized, which proved to be less-toxic than previously synthesized hits on the human HepG2 cell line, but did not display significant antiplasmodial activity. A brief Structure-Activity Relationship (SAR) evaluation shows that a more restricted conformational freedom is probably necessary for providing antiplasmodial activity.

## 1. Introduction

The quinazoline ring is an important molecular scaffold whose derivatives display a wide variety of pharmacological properties. Indeed, they were used as potent tyrosine kinase and cellular phosphorylation inhibitors [1], and they also act as ligands of benzodiazepine and GABA receptors in the central nervous system (CNS) [2] or as DNA binders [3]. Some of them exhibit remarkable activity as anticancer [4,5,6], antiviral [6,7,8], antibacterial [9] and anti-TB agents [10,11]. Moreover, popular drugs containing the quinazoline unit are available on the market. For example, erlotinib and gefitinib are used as epidermal growth factor receptor inhibitors in the treatment of several types of tumors [12], especially lung cancer [13,14]. In the other hand, prazosin acts as an *α*-adrenergic blocker and is used to treat high blood pressure [15]. In continuation of our research program centered on the design and synthesis of original molecules with pharmacological properties [16,17,18,19], our group recently described the preparation of new 4-substituted-2-trichloromethylquinazoline derivatives which exhibit original *in vitro* antiplasmodial properties [20,21,22,23,24]. The promising results displayed by these recent studies prompted us to synthesize new derivatives in this 2-trichloromethylquinazoline series in order to identify both more potent antiplasmodial derivatives and less toxic ones, in order to increase the selectivity index (SI). With this objective, we focused on the synthesis of *N*-(quinazolin-4-yl)benzenesulfonamides, taking into account that the sulfonamide group is well-known for its pharmaceutical interest in the field of anti-infectious substances. 

## 2. Results and Discussion

### 2.1. Synthesis

Earlier works described the synthesis of *N*-(quinazolin-4-yl)benzenesulfonamides by nucleophilic substitution of 4-methoxyquinazoline by the sulfanilamide anion [9], by the construction of the quinazoline core from *N*-arylimidines and *N*-tosylisocyanodichloride [25] or by reacting *p*-toluene-sulfonylisothiocyanate with 2-arylquinazoline-4(*3H*)-thione [26]. 

**Scheme 1:**
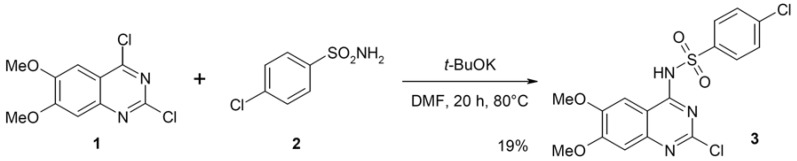
Synthesis of sulfonamide **3** starting from 4-chloroquinazoline **1**.

More recent publications report the access to these sulfonamides from the readily available 4-chloroquinazoline derivatives. Aryl-sulfonamides **2** in the presence of bases such as sodium hydride or potassium *t*-butoxide underwent nucleophilic substitution of 4-chloroquinazoline **1** and afforded the desired compound **3** in limited yields [27,28] (Scheme 1). 

**Scheme 2:**
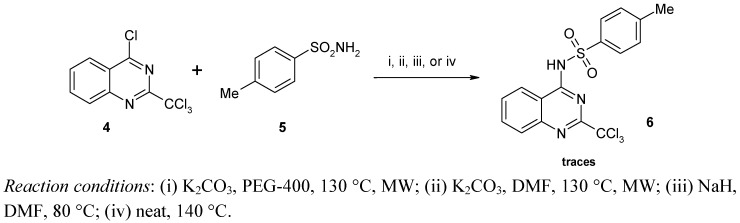
Synthesis of sulfonamide **6** starting from 4-chloro-2-trichloromethylquinazoline (**4**).

It is worth mentioning that this reaction was never attempted starting from 4-chloro-2-trichloro-methylquinazoline [29]. Although the yields obtained with this strategy remained low, we tried to perform this reaction using *p*-toluenesulfonamide. Unfortunately, in our hands, whatever the operating conditions applied only traces of product were detected with LC-MS (Scheme 2).

**Scheme 3:**
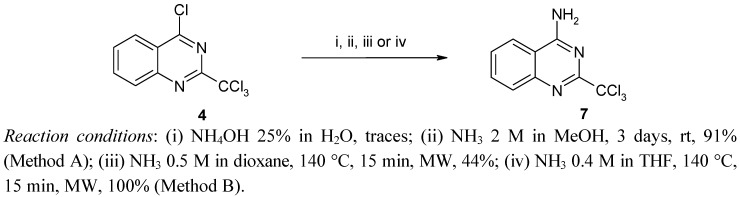
Synthesis of 4-amino-2-trichloromethylquinazoline (**7**).

Facing these failures, we chose to reverse the strategy by reacting readily available sulfonyl chlorides with 4-amino-2-trichloromethylquinazoline (**7**). We therefore investigated first of all the optimal synthesis of the key intermediate derivative **7** (Scheme 3).

The reaction of 4-chloro-2-trichloromethylquinazoline (**4**) with 25% aqueous ammonium hydroxide led to the predominant formation of 2-trichloromethylquinazolin-4(*3H*)-one [23] accompanied by traces of the expected amino derivative **7**, so it appeared that it was necessary to operate under anhydrous conditions to prevent hydrolysis. Thus, the use of a solution of 2N ammonia in dry methanol afforded the expected amino derivative **7** in 71% yield after 24 h at room temperature. Increasing the time of the reaction up to 72 h increased the yield to 91% (Method A), while elevating the temperature with a view to shortening reaction time was detrimental and led mainly to the formation of 4-methoxy-2-trichloromethylquinazoline. In order to prevent this side reaction, due to the nucleophilic character of methanol, the amination must be conducted in a solvent inert towards the starting material **4**. In order to shorten the reaction time we investigated the benefit of using sealed vials under microwave irradiation. Thus, under such conditions, the use of ammonia in dioxane (0.5 M, 3 equiv.) afforded **7** in only 44% yield at 140 °C. Finally, we found that heating **4** under the same conditions in THF (0.4 M, 3 equiv.) led to **7** in quantitative yield after only 15 min (Method B) which is not surprising given the known ability of THF to absorb microwaves. 

**Scheme 4:**
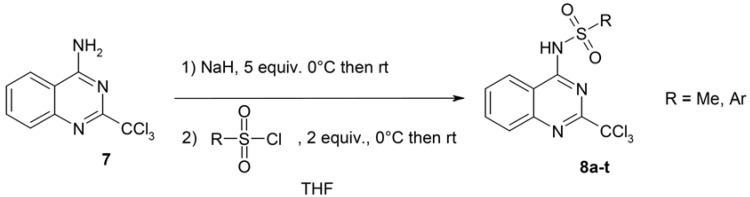
Synthesis of *N*-(2-trichloromethylquinazolin-4-yl)sulfonamides **8**.

Having secured good access to this key intermediate 4-amino-2-trichloromethylquinazoline (**7**), we next synthesized the target sulfonamides using variously substituted sulfonyl chlorides (Scheme 4) with a view to further study the influence of the substituent nature on the biological properties. Amino derivative **7** was reacted with an excess of sodium hydride in THF. The so-formed anion was then trapped with sulfonyl chloride. The resulting sulfonamides **8** were thus obtained in good yields (Table 1) without requiring any chromatographic purification (except for compound **8t**).

**Table 1:** Reaction yields and biological evaluation for sulfonamides **8a**–**t**.

Entry	R-	Product	Yield %	CC_50_ (µM) ^a^	IC_50_ P *f*K1 (µM) ^b^
1	Ph-	**8a**	62	82.0	>10 *
2	4-Me-Ph-	**8b**	51	68.7	>10 *
3	4-MeO-Ph-	**8c**	56	102.3	>10 *
4	4-CN-Ph-	**8d**	73	73.8	>10 *
5	4-Cl-Ph	**8e**	50	71.1	>10 *
6	4-Br-Ph-	**8f**	60	57.4	>10 *
7	4-F-Ph-	**8g**	66	69.5	>10 *
8	3-F-Ph-	**8h**	53	106.4	>10 *
9	2-F-Ph-	**8i**	60	136.1	>10 *
10	4-NO_2_-Ph-	**8j**	91	88.3	>10 *
11	3-NO_2_-Ph-	**8k**	56	119.8	>10 *
12	2-NO_2_-Ph-	**8l**	59	126.0	>10 *
13	4-CF_3_-Ph-	**8m**	88	49.0	>10 *
14	3-CF_3_-Ph-	**8n**	100	75.1	>10 *
15	2-CF_3_-Ph-	**8o**	50	73.8	>10 *
16	Biphenyl-4-yl	**8p**	23	38.2	>10 *
17	Napht-2-yl-	**8q**	43	48.8	>10 *
18	Napht-1-yl-	**8r**	61	47.8	>10 *
19	Thiophen-2-yl-	**8s**	43	121.1	>10 *
20	Methyl-	**8t**	18	241.2	>10 *
Doxorubicine ^a^				*0.2*	*-*
Chloroquine ^b^				*30*	*0.6*
Doxycycline ^b^				*20*	*6.0*

^a^ Doxorubicine was used as a cytotoxic reference-drug; ^b^ chloroquinine and doxycycline were used as antimalarial reference-drugs; * Tested compounds did not show significant antiplasmodial activity at the highest tested concentration (10 µM).

Our efficient two-step methodology afforded the expected compounds in a 50–100% range yield except for the biphenyl-4-yl-, napht-2-yl, thiophen-2-yl and the methyl derivatives (compounds **8p**, **8q**, **8s** and **8t**, respectively, entries 16, 17, 19 and 20) which were not obtained from simple benzenesulfonyl chlorides. One the one hand, the electron withdrawing or donating character of the substituent of benzenesulfonyl chlorides did not affect appear to notably the reaction yield. However, entries 4, 10, 13 and 14 corresponding to electron withdrawing groups are the ones which gave the best yields. On the other hand, the lowest reaction yields are attributable to the purification process (namely the high solubility of molecules **8p**–**t** in CH_2_Cl_2_).

### 2.2. Biological Evaluation and Structure-Activity Relationship (SAR) Study

Following previously reported experimental procedures [22,23], the *in vitro* antiplasmodial activity toward the multi-resistant W2 or K1 *Plasmodium falciparum* strains was measured by determining the IC_50_ (inhibition concentration 50% in µM) and comparing them with two commercial reference-drugs: chloroquine and doxycycline. The cytotoxicity was measured toward the human HepG2 cell line by determining the CC_50_ (cytotoxic concentration 50% in µM) and comparing them with doxorubicine used as a cytotoxic reference-drug. Concerning the biological evaluation, this series showed excellent solubility in biological media, contrary to the 4-thiophenoxy- series [22] whose cytotoxic evaluation was limited by a lack of solubility (Table 2, entry 4).

**Table 2:** Antiplasmodial and cytotoxicity evaluation in 2-trichloromethylquinazoline series.

Entry	Structure	CC_50_ HepG2 (µM)	IC_50_ *P. falc*. (µM)	SI ^a^
**1**	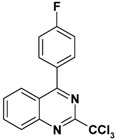	>125	2.5	>50
**2**	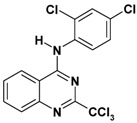	16	0.4	40
**3**	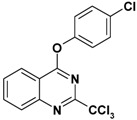	50	1.1	45
**4**	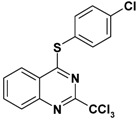	>25	0.9	>28
**5**	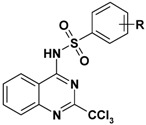	49–136	>10	nd
Doxorubicine ^b^		0.2	-	-
Chloroquine ^c^		30	0.6	
Doxycycline ^c^		20	6.0	

^a^ Selectivity index (SI) was calculated according to the formula: SI = CC_50_/IC_50_; ^b^ Doxorubicine was used as a cytotoxic reference-drug; ^c^ Chloroquinine and Doxycycline were used as antimalarial reference-drugs.

Unfortunately, contrary to the previously synthesized series (Table 2, entries 1–4), the sulfonamide derivatives (Table 1 and Table 2, entry 5) did not display significant antiplasmodial activity with IC_50_ values >10 µM. The Structure-Activity Relationship study seems to indicate that the introduction of a sulfonamide group at position 4 of the quinazoline ring leads to a larger gap between the phenyl substituent and the quinazoline ring. This structural change added a more important conformational freedom and reduced antiplasmodial activity. The most potent antiplasmodial molecule previously synthesized (Table 2, entry 2) displayed slight cytotoxicity (16 µM) on the HepG2 human cell line which limits its selectivity index to 40. Concerning the sulfonamide derivatives, apart from the biphenyl derivative **8p** (Table 1), this series appeared less-toxic than previously synthesized hits on the same cell line. 

**Figure 1:**
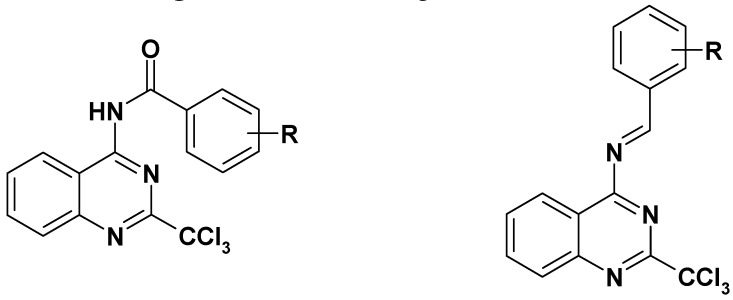
Novel investigated derivatives.

Thus, taking advantage of this brief SAR evaluation, two new 4-substituted quinazoline series presenting a more restricted conformational freedom (Figure 1) could be prepared by using the synthetic approach presented herein, in order to identify new antiplasmodial hits. 

## 3. Experimental

### 3.1. General Procedures

Commercial reagents were used as received without additional purification. Melting points were determined on a Köfler melting point apparatus and are uncorrected. Elemental analyses and HRMS were carried out at the Spectropole, Faculté des Sciences et Techniques de Saint-Jérôme, Marseille, France. NMR spectra were recorded on a Bruker ARX 200 spectrometer at the Faculté de Pharmacie de Marseille (200 MHz ^1^H-NMR: reference CHCl_3_ δ = 7.26, DMSO-*d*_6_ δ = 2.50 and 50 MHz ^13^C: reference CHCl_3_ δ = 76.9, DMSO-*d*_6_ δ = 39.5). Solvents were dried by conventional methods. The following adsorbent was used for column chromatography: silica gel 60 (Merck, particle size 0.063–0.200 mm, 70–230 mesh ASTM). TLC was performed on 5 cm × 10 cm aluminium plates coated with silica gel 60F-254 (Merck) in an appropriate eluent. Visualization was made with ultraviolet light (234 nm). HRMS spectra were recorded on QStar Elite (Applied Biosystems SCIEX) spectrometer. PEG was the matrix for HRMS. The experimental exact mass was given for the ion which has the maximum isotopic abundance. Purity of synthetized compounds was checked with LC-MS analyses which were realized at the Faculty of Pharmacy of Marseille with a Thermo Scientific Accela High Speed LC System^®^ coupled with a single quadrupole mass spectrometer Thermo MSQ Plus^®^. The RP-HPLC column used is a Thermo Hypersil Gold^®^ 50 × 2.1 mm (C18 bounded), with particles of 1.9 µm diameter. The volume of sample injected on the column was 1 µL. The chromatographic analysis, total duration of 8 min, is made with the gradient of following solvents: t = 0 min, water/methanol 50/50; 0 < t < 4 min, linear increase in the proportion of water to a ratio water/methanol 95/5; 4 < t < 6 min, water/methanol 95/5; 6 < t < 7 min, linear decrease in the proportion of water to return to a ratio 50/50 water/methanol; 6 < t < 7 min, water/methanol 50/50. The water used was buffered with 5 mM ammonium acetate. The retention times (Tr) of the molecules analyzed are indicated in min. The microwave reactions were performed using a Biotage Initiator Microwave oven using 10–20 mL sealed vials; temperatures were measured with an IR-sensor and reaction times given as hold times. The preparation of 4-chloro-2-trichloromethylquinazoline (**4**) was achieved as described in the literature [29].

### 3.2. Preparation of 4-Amino-2-trichloromethylquinazoline (***7***)

*Method (A)* To a stirred solution of 4-chloro-2-trichloromethylquinazoline (**4**, 2.0 g, 7.1 mmol) in dry THF (10 mL) under nitrogen at rt, was added ammonia solution in methanol (2.0 M, 10.6 mL, 21.3 mmol, 3 equiv.). The resulting mixture was stirred at this temperature for 3 days, and then, the volatiles were removed under vacuum. The residue was triturated with dichloromethane and filtered to give **7** as a white solid (1.7 g, 91%).

*Method (B)* In a microwave vial equipped with a magnetic stir bar, 4-chloro-2-trichloromethylquinazoline (**4**, 700 mg, 2.48 mmol) and ammonia solution in THF (0.4 M, 18.6 mL, 7.45 mmol, 3 equiv.). The vial was capped and the suspension was then heated at 140 °C for 15 min (8 bar). The volatiles were removed under vacuum. The residue was poured into EtOAc (100 mL) and extracted two times with brine (30 mL). The organic layer was dried with Na_2_SO_4_, filtered and evaporated to afford **2** as white solid (650 mg, 100%). M.p. 199 °C. ^1^H-NMR (DMSO-*d*_6_): δ 8.33–8.27 (m, 3H), 7.90–7.77 (m, 2H), 7.65–7.57 (m, 1H). ^13^C-NMR (DMSO-*d*_6_): δ 163.2, 160.9, 148.8, 134.1, 128.3, 127.5, 123.9, 113.4, 98.4. LC-MS (ESI) Tr 2.37 min, *m/z* [M+H]^+^ Calcd: 261.96, Found: 262.03. Anal. calcd. for C_9_H_6_Cl_3_N_3_: C, 41.18; H, 2.30; N, 16.01. Found: C, 41.43; H, 2.31; N, 16.29. 

### 3.3. General Procedure for the Preparation of Compounds ***8a*** to ***8t***

To a slurry of 60% sodium hydride in oil (152 mg, 3.81 mmol, 5 equiv.) in dry THF (4 mL) at 0 °C was slowly added 4-amino-2-trichloromethylquinazoline (**7**, 200 mg, 0.76 mmol, 1 equiv.). After 30 min of stirring at rt, the mixture was cooled again and the appropriate sulfonyl chloride (1.52 mmol, 2 equiv.) was added portionwise. After this addition, the reaction was then stirred at rt until the disappearance of starting material. The excess of NaH was hydrolyzed with ice at 0 °C. The mixture was extracted with EtOAc and washed three times with brine. The organic layer was dried with Na_2_SO_4_, filtered and evaporated. The crude residue was triturated in dichloromethane and filtered to give the corresponding sulfonamide **8**.

*N-(2-Trichloromethylquinazolin-4-yl)benzenesulfonamide* (**8a**). White solid (62%). M.p. 200 °C. ^1^H-NMR (DMSO-*d*_6_): δ 8.27 (d, *J =* 8.0 Hz, 1H), 8.02 (t, *J =* 3.5 Hz, 2H), 7.75–7.60 (m, 2H), 7.48 (t, *J =* 7.0 Hz, 1H), 7.38–7.35 (m, 3H). ^13^C-NMR (DMSO-*d*_6_): δ 163.1, 160.3, 148.6, 144.8, 132.5, 130.0, 127.8, 127.5, 127.1, 126.4, 125.4, 119.1, 98.9. LC-MS (ESI) Tr 1.36 min, *m/z* [M+H]^+^ Calcd: 401.96, Found: 401.96. HRMS (ESI+) *m/z* 423.9454 [M+Na]^+^, calcd. for C_15_H_10_N_3_O_2_SCl_3_: 423.9452 [M+Na]^+^.

*4-Methyl-N-(2-trichloromethylquinazolin-4-yl)benzenesulfonamide* (**8b**). White solid (51%). M.p. > 260 °C. ^1^H-NMR (DMSO-*d*_6_): δ 8.24 (d, *J =* 7.8 Hz, 1H), 7.93 (d, *J =* 7.6 Hz, 2H), 7.69–7.58 (m, 2H); 7.45 (t, *J =* 7.6 Hz, 1H), 7.15 (d, *J =* 7.6 Hz, 2H), 2.27 (s, 3H). ^13^C-NMR (DMSO-*d*_6_): δ 163.2, 160.5, 148.6, 142.2, 139.6, 132.3; 127.9, 127.8, 127.0, 126.2, 125.4, 119.4, 99.1; 20.9. LC-MS (ESI) Tr 1.98 min, *m/z* [M−H]^−^ Calcd: 413.97, Found: 414.35. HRMS (ESI+) *m/z* 437.9607 [M+Na]^+^, calcd. for C_16_H_12_N_3_O_2_SCl_3_: 437.9608 [M+Na]^+^.

*4-Methoxy-N-(2-trichloromethylquinazolin-4-yl)benzenesulfonamide* (**8c**). White solid (56%). M.p. 196 °C. ^1^H-NMR (DMSO-*d*_6_): δ 8.23 (d, *J =* 7.8 Hz, 1H), 7.99 (d, *J =* 8.4 Hz, 2H), 7.69–7.61 (m, 2H); 7.45 (t, *J =* 7.2 Hz, 1H), 6.87 (d, *J =* 8.4 Hz, 2H), 3.73 (s, 3H). ^13^C-NMR (DMSO-*d*_6_): δ 163.1, 160.5, 148.6, 137.2, 132.2, 129.8; 127.0, 126.2, 125.4, 119.4, 112.5, 99.1; 55.3. LC-MS (ESI) Tr 1.29 min, *m/z* [M+H]^+^ Calcd: 431.97, Found: 431.90. HRMS (ESI+) *m/z* 453.9559 [M+Na]^+^, calcd. for C_16_H_12_N_3_O_3_SCl_3_: 453.9557 [M+Na]^+^.

*4-Cyano-N-(2-trichloromethylquinazolin-4-yl)benzenesulfonamide* (**8d**). Beige solid (73%). M.p. 204 °C. ^1^H-NMR (DMSO-*d*_6_): δ 8.34 (d, *J =* 7.8 Hz, 1H), 8.15 (d, *J =* 8.2 Hz, 2H), 7.87 (d, *J =* 8.4 Hz, 2H), 7.82–7.67 (m, 2H); 7.55 (dt, *J =* 1.5 Hz, *J =* 8.2 Hz, 1H). ^13^C-NMR (DMSO-*d*_6_): δ 162.6, 159.8, 148.8, 148.6, 133.2, 132.1, 128.4; 127.4, 127.1, 125.2, 118.4, 118.3, 112.8, 98.3. LC-MS (ESI) Tr 1.19 min, *m/z* [M−H]^−^ Calcd: 424.95, Found: 425.39. HRMS (ESI+) *m/z* 448.9406 [M+Na]^+^, calcd. for C_16_H_9_N_4_O_2_SCl_3_: 448.9404 [M+Na]^+^.

*4-Chloro-N-(2-trichloromethylquinazolin-4-yl)benzenesulfonamide* (**8e**). White solid (50%). M.p. > 260 °C. ^1^H-NMR (DMSO-*d*_6_): δ 8.25 (d, *J =* 7.6 Hz, 1H), 7.99 (d, *J =* 8.2 Hz, 2H), 7.71–7.63 (m, 2H); 7.48–7.39 (m, 3H). ^13^C-NMR (DMSO-*d*_6_): δ 163.2, 160.2, 148.6, 144.0, 134.5, 132.5, 129.6; 127.5, 127.0, 126.4, 125.3, 119.1, 98.8. LC-MS (ESI) Tr 2.54 min, *m/z* [M+H]^+^ Calcd: 435.92, Found: 435.85. HRMS (ESI+) *m/z* 437.9211 [M+H]^+^, calcd. for C_15_H_9_N_3_O_2_SCl_4_: 436.9141. 

*4-Bromo-N-(2-trichloromethylquinazolin-4-yl)benzenesulfonamide* (**8f**). White solid (60%). M.p. > 260 °C. ^1^H-NMR (DMSO-*d*_6_): δ 8.25 (d, *J =* 7.8 Hz, 1H), 7.92 (d, *J =* 8.2 Hz, 2H), 7.72–7.45 (m, 5H). ^13^C-NMR (DMSO-*d*_6_): δ 163.4, 160.3, 148.6, 144.5, 132.5, 130.5, 129.9; 127.1, 126.4, 125.4, 123.3, 119.3, 98.9. LC-MS (ESI) Tr 2.60 min, *m/z* [M+H]^+^ Calcd: 479.87, Found: 480.52. HRMS (ESI+) *m/z* 503.8534 [M+Na]^+^, calcd. for C_15_H_9_BrN_3_O_2_SCl_3_: 503.8532 [M+Na]^+^.

*4-Fluoro-N-(2-trichloromethylquinazolin-4-yl)benzenesulfonamide* (**8g**). White solid (66%). M.p. > 260 °C. ^1^H-NMR (DMSO-*d*_6_): δ 8.25 (d, *J =* 8.0 Hz, 1H), 8.10–8.03 (m, 2H), 7.75–7.60 (m, 2H), 7.48 (t, *J =* 7.1 Hz, 1H), 7.17 (t, *J =* 8.9 Hz, 2H). ^13^C-NMR (DMSO-*d*_6_): δ 165.4, 163.4, 160.3, 148.6, 141.5 (d, *J =* 2.9 Hz), 132.4, 130.5 (d, *J =* 8.8 Hz), 127.1, 126.4, 125.4, 119.3, 114.3 (d, *J =* 21.8 Hz), 98.9. LC-MS (ESI) Tr 1.81 min, *m/z* [M+H]^+^ Calcd: 419.95, Found: 419.79. HRMS (ESI+) *m/z* 419.9534 [M+H]^+^, calcd. for C_15_H_9_N_3_O_2_SCl_3_F: 418.9465.

*3-Fluoro-N-(2-trichloromethylquinazolin-4-yl)benzenesulfonamide* (**8h**). White solid (53%). M.p. 194 °C. ^1^H-NMR (DMSO-*d*_6_): δ 8.30 (d, *J =* 8.0 Hz, 1H), 7.88 (d, *J =* 9.0 Hz, 1H), 7.77–7.65 (m, 3H), 7.53 (t, *J =* 7.8 Hz, 1H), 7.44–7.38 (m, 1H), 7.25 (t, *J =* 8.2 Hz, 1H). ^13^C-NMR (DMSO-*d*_6_): δ 163.6, 162.8, 160.1, 158.8, 148.6, 146.8 (d, *J =* 6.5Hz), 132.9, 129.9 (d, *J =* 7.6 Hz), 127.1 (d, *J =* 21.3 Hz), 125.3, 123.4 (d, *J =* 2.9 Hz), 118.6, 117.3 (d, *J =* 20.9 Hz), 115.4 (d, *J =* 23.4 Hz), 98.5. LC-MS (ESI) Tr 1.69 min, *m/z* [M+H]^+^ Calcd: 419.95, Found: 419.81. HRMS (ESI+) *m/z* 441.9359 [M+Na]^+^, calcd. for C_15_H_9_N_3_O_2_SCl_3_F: 441.9357 [M+Na]^+^.

*2-Fluoro-N-(2-trichloromethylquinazolin-4-yl)benzenesulfonamide* (**8i**). White solid (60%). M.p. 188 °C. ^1^H-NMR (DMSO-*d*_6_): δ 8.28 (d, *J =* 8.0 Hz, 1H), 8.06 (dt, *J =* 1.5 Hz, *J =* 7.6 Hz, 1H), 7.78–7.61 (m, 2H), 7.50 (dt, *J =* 1.6 Hz, *J =* 7.6 Hz, 1H), 7.22 (t, *J =* 7.5 Hz, 1H), 7.07 (t, *J =* 8.9 Hz, 1H). ^13^C-NMR (DMSO-*d*_6_): δ 163.4, 160.3, 158.0 (d, *J =* 248.8 Hz), 148.7, 133.1 (d, *J =* 15.3 Hz), 132.6, 132.0 (d, *J =* 8.1 Hz), 131.8, 127.1, 126.4, 125.4, 123.5 (d, *J =* 3.6 Hz), 119.0; 115.7 (d, *J =* 21.9 Hz), 98.5. LC-MS (ESI) Tr 1.25 min, *m/z* [M+H]^+^ Calcd: 419.95, Found: 419.80. HRMS (ESI+) *m/z* 441.9360 [M+Na]^+^, calcd. for C_15_H_9_N_3_O_2_SCl_3_F: 441.9357 [M+Na]^+^.

*4-Nitro-N-(2-trichloromethylquinazolin-4-yl)benzenesulfonamide* (**8j**). Yellow solid (91%). M.p. 250 °C. ^1^H-NMR (DMSO-*d*_6_): δ 8.30–8.20 (m, 5H), 7.78–7.71 (m, 2H), 7.51 (d, *J =* 7.2 Hz, 1H). ^13^C-NMR (DMSO-*d*_6_): δ 163.6, 160.0, 151.6, 148.6, 148.0, 132.7, 128.9; 127.1, 126.6, 125.4, 123.0, 119.1, 98.6. LC-MS (ESI) Tr 1.87 min, *m/z* [M−H]^−^ Calcd: 444.94, Found: 445.16. HRMS (ESI+) *m/z* 468.9306 [M+Na]^+^, calcd. for C_15_H_9_N_4_O_4_SCl_3_: 468.9302 [M+Na]^+^.

*3-Nitro-N-(2-trichloromethylquinazolin-4-yl)benzenesulfonamide* (**8k**). Beige solid (56%). M.p. 204 °C. ^1^H-NMR (DMSO-*d*_6_): δ 8.76 (s, 1H), 8.30–8.21 (m, 3H), 7.74–7.61 (m, 3H), 7.51 (t, *J =* 7.6 Hz, 1H). ^13^C-NMR (DMSO-*d*_6_): δ 163.5, 160.1, 148.5, 147.3, 147.1, 133.6, 132.7; 129.6, 127.2, 126.7, 125.4, 124.6, 123.2, 119.1, 98.5. LC-MS (ESI) Tr 1.40 min, *m/z* [M−H]^−^ Calcd: 444.94, Found: 445.17. HRMS (ESI+) *m/z* 468.9303 [M+Na]^+^, calcd. for C_15_H_9_N_4_O_4_SCl_3_: 468.9302 [M+Na]^+^.

*2-Nitro-N-(2-trichloromethylquinazolin-4-yl)benzenesulfonamide* (**8l**). Beige solid (59%). M.p. > 260 °C. ^1^H-NMR (DMSO-*d*_6_): δ 8.34–8.25 (m, 2H), 7.75–7.53 (m, 6H). ^13^C-NMR (DMSO-*d*_6_): δ 163.7, 160.2, 148.7, 147.9, 137.3, 132.7, 131.8; 131.0, 130.7, 127.1, 126.5, 125.5, 122.2, 119.1, 98.5. LC-MS (ESI) Tr 1.16 min, *m/z* [M−H]^−^ Calcd: 444.94, Found: 445.25. HRMS (ESI+) *m/z* 468.9303 [M+Na]^+^, calcd. for C_15_H_9_N_4_O_4_SCl_3_: 468.9302 [M+Na]^+^.

*N-(2-trichloromethylquinazolin-4-yl)-4-(trifluoromethyl)benzenesulfonamide* (**8m**). White solid (88%). M.p. 166 °C. ^1^H-NMR (DMSO-*d*_6_): δ 8.28 (d, *J =* 7.6 Hz, 1H), 8.13 (d, *J =* 7.2 Hz, 2H), 7.74–7.50 (m, 5H). ^13^C-NMR (DMSO-*d*_6_): δ 163.5, 160.2, 149.4, 148.6, 132.6, 129.8 (q, *J =* 31.8 Hz), 128.3, 127.1, 126.5, 125.4, 124.8 (q, *J =* 3.7 Hz), 124.0 (q, *J =* 271.0 Hz), 119.5, 98.6. LC-MS (ESI) Tr 2.80 min, *m/z* [M+H]^+^ Calcd: 469.94, Found: 469.82. HRMS (ESI+) *m/z* 491.9328 [M+Na]^+^, calcd. for C_16_H_9_F_3_N_3_O_2_SCl_3_: 491.9325 [M+Na]^+^.

*N-(2-trichloromethylquinazolin-4-yl)-3-(trifluoromethyl)benzenesulfonamide* (**8n**). White solid (100%). M.p. 255 °C. ^1^H-NMR (DMSO-*d*_6_): δ 8.44 (d, *J =* 8.0 Hz, 1H), 8.33–8.29 (m, 2H), 7.91–7.82 (m, 3H); 7.76 (d, *J =* 8.0 Hz, 1H), 7.70–7.62 (m, 1H). ^13^C-NMR (DMSO-*d*_6_): δ 160.6, 159.3, 148.7, 143.9, 134.1, 131.5, 130.0, 129.1 (q, *J =* 32.2 Hz), 128.4 (q, *J =* 3.5 Hz), 128.0, 127.8, 124.7, 124.4 (q, *J =* 4.1 Hz), 123.7 (q, *J =* 271 Hz), 116.6, 97.5. LC-MS (ESI) Tr 2.53 min, *m/z* [M+H]^+^ Calcd: 469.94, Found: 469.76. HRMS (ESI+) *m/z* 469.9502 [M+H]^+^, calcd. for C_16_H_9_F_3_N_3_O_2_SCl_3_: 468.9433.

*N-(2-trichloromethylquinazolin-4-yl)-2-(trifluoromethyl)benzenesulfonamide* (**8o**). White solid (50%). M.p. 205 °C. ^1^H-NMR (DMSO-*d*_6_): δ 8.71 (d, *J =* 8.2 Hz, 1H), 8.51 (d, *J =* 7.2 Hz, 1H), 8.04–7.82 (m, 6H). ^13^C-NMR (DMSO-*d*_6_): δ 158.2, 158.1, 148.5, 138.8, 135.4, 133.4, 133.3, 132.8, 129.3, 128.2, 128.0 (q, *J =* 6.3 Hz), 125.7 (q, *J =* 32.8 Hz), 124.1, 122.9 (q, *J =* 272.4 Hz), 114.2, 96.3. LC-MS (ESI) Tr 2.17 min, *m/z* [M+H]^+^ Calcd: 469.94, Found: 469.76. HRMS (ESI+) *m/z* 491.9327 [M+Na]^+^, calcd. for C_16_H_9_F_3_N_3_O_2_SCl_3_: 491.9325 [M+Na]^+^.

*N-(2-trichloromethylquinazolin-4-yl)biphenyl-4-sulfonamide* (**8p**). White solid (23%). M.p. 210 °C. ^1^H-NMR (DMSO-*d*_6_): δ 8.29 (d, *J =* 7.8 Hz, 1H), 8.11 (d, *J =* 8.2 Hz, 2H), 7.73–7.65 (m, 6H), 7.62–7.36 (m, 4H). ^13^C-NMR (DMSO-*d*_6_): δ 163.0, 160.3, 148.6, 143.8, 141.8, 139.6, 132.5, 129.0, 128.5, 127.8, 127.1, 126.9, 126.5, 125.9, 125.4, 119.1, 98.9. LC-MS (ESI) Tr 3.06 min, *m/z* [M−H]^−^ Calcd: 475.99, Found: 476.30. HRMS (ESI+) *m/z* 499.9762 [M+Na]^+^, calcd. for C_21_H_14_N_3_O_2_SCl_3_: 499.9765 [M+Na]^+^.

*N-(2-trichloromethylquinazolin-4-yl)naphtalene-2-sulfonamide* (**8q**). Beige solid (43%). M.p. 206 °C. ^1^H-NMR (DMSO-*d*_6_): δ 8.65 (s, 1H), 8.28 (d, *J =* 8.0 Hz, 1H), 8.00 (d, *J =* 8.4 Hz, 2H), 7.85 (d, *J =* 8.5 Hz, 2H), 7.60–7.48 (m, 5H). ^13^C-NMR (DMSO-*d*_6_): δ 163.3, 160.4, 148.6, 142.3, 133.4, 132.4, 131.8, 128.8, 128.0, 127.5, 127.1, 126.4, 126.3, 125.4, 124.6, 119.3, 98.8. LC-MS (ESI) Tr 2.39 min, *m/z* [M+H]^+^ Calcd: 451.97, Found: 451.79. HRMS (ESI+) *m/z* 451.9785 [M+H]^+^, calcd. for C_19_H_12_N_3_O_2_SCl_3_: 450.9716.

*N-(2-trichloromethylquinazolin-4-yl)naphtalene-1-sulfonamide* (**8r**). White solid (61%). M.p. > 260 °C. ^1^H-NMR (DMSO-*d*_6_): δ 8.86 (d, *J =* 7.8 Hz, 1H), 8.43–8.33 (m, 2H), 7.95–7.84 (m, 2H), 7.68–7.42 (m, 6H). ^13^C-NMR (DMSO-*d*_6_): δ 163.4, 160.3, 148.7, 141.0, 133.3, 132.3, 130.6, 129.3, 128.5, 128.0, 127.0, 126.4, 126.3, 126.1, 125.5, 125.3, 124.5, 119.1, 98.7. LC-MS (ESI) Tr 2.32 min, *m/z* [M+H]^+^ Calcd: 451.97, Found: 451.64. HRMS (ESI+) *m/z* 473.9608 [M+Na]^+^, calcd. for C_19_H_12_N_3_O_2_SCl_3_: 473.9608 [M+Na]^+^.

*N-(2-trichloromethylquinazolin-4-yl)thiophene-2-sulfonamide* (**8s**). Beige solid (43%). M.p. 191 °C. ^1^H-NMR (DMSO-*d*_6_): δ 8.23 (d, *J =* 8.0 Hz, 1H), 7.83 (d, *J =* 3.0 Hz, 1H), 7.77–7.62 (m, 2H), 7.57–7.45 (m, 2H), 6.97 (t, *J =* 4.2 Hz, 1H). ^13^C-NMR (DMSO-*d*_6_): δ 163.5, 160.5, 148.7, 146.7, 146.9, 132.5, 130.6, 128.8, 127.1, 126.4, 126.1, 125.4, 119.4, 99.0. LC-MS (ESI) Tr 1.07 min, *m/z* [M+H]^+^ Calcd: 407.91, Found: 407.86. HRMS (ESI+) *m/z* 429.9014 [M+Na]^+^, calcd. for C_13_H_8_N_3_O_2_S_2_Cl_3_: 429.9016 [M+Na]^+^.

*N-(2-trichloromethylquinazolin-4-yl)methanesulfonamide* (**8t**). White solid (18%). M.p. 186 °C. ^1^H-NMR (DMSO-*d*_6_): δ 8.47 (d, *J =* 8.0 Hz, 1H), 7.97–7.92 (m, 2H), 7.72 (t, *J =* 6.2 Hz, 1H), 3.67 (s, 3H). ^13^C-NMR (DMSO-*d*_6_): δ 158.6, 158.2, 149.1, 135.5, 129.3, 128.7, 123.9, 113.7, 97.0, 42.1. LC-MS (ESI) Tr 0.82 min, *m/z* [M+H]^+^ Calcd: 339.94, Found: 339.89. HRMS (ESI+) *m/z* 339.9475 [M+H]^+^, calcd. for C_10_H_8_N_3_O_2_SCl_3_: 338.9403.

## 4. Conclusions

A series of 20 quinazolines was synthetized by an efficient two-step strategy. Firstly, 4-amino-2-trichloromethylquinazoline (**7**) was prepared in quantitative yield from the corresponding 4-chloro derivative under microwave irradiation. Then, the key amino intermediate **7** was reacted with various sulfonyl chlorides in the presence of NaH, leading to the expected sulfonamides **8**. Synthesized compounds were highly soluble in biological media, less-toxic toward the human HepG2 cell line than previously identified hits but did not display significant *in vitro* antiplasmodial activity.
